# Objective test findings in patients with chronic eye symptoms seeking care at an outpatient neck center: ligamentous cervical instability etiology?

**DOI:** 10.3389/fneur.2025.1576315

**Published:** 2025-09-22

**Authors:** Ross A. Hauser, Morgan Griffiths, Ashley Watterson, Danielle Matias, Benjamin Rawlings

**Affiliations:** ^1^Caring Medical Florida, Commerce Center Court, Fort Myers, FL, United States; ^2^Independent Researcher, Fort Myers, FL, United States; ^3^Division of Behavioral and Organizational Sciences, Claremont Graduate University, Claremont, CA, United States

**Keywords:** intraocular pressure, intracranial pressure, optic nerve sheath diameter, ligamentous cervical instability, internal jugular vein, cerebrospinal fluid, depth of curve

## Abstract

**Purpose:**

To examine the incidence and potential associations between eye symptoms and objective test findings in patients reporting to an outpatient neck center without known preexisting conditions.

**Methods:**

Consecutive patients between January 1 and June 30, 2022, reporting at least 1 of 6 eye symptoms (light sensitivity, blurry vision, eye pain/pressure, vision changes, seeing flashes of light, eye tearing) without known etiology underwent these tests: pupillometry, tonometry, ultrasound of carotid sheath and optic nerve sheath, and digital motion x-ray (videofluoroscopy) and upright cone beam computed tomography scan of cervical spine.

**Results:**

The analysis included 203/234 consecutive patients. Elevated optic nerve sheath diameter (total >12.2 mm) was documented in 98% (199/203). Supine cervical ultrasound revealed 99.5% (202/203) with internal jugular vein narrowing at C1: total internal jugular vein cross-sectional area <180 mm^2^. Mean internal jugular vein cross-sectional area at C1 was significantly higher with cervical orthotic Denneroll^®^ (+35.76 mm, *p* < 0.05). Some 95.6% evidenced vagus nerve degeneration (total cross-sectional area <4.2 mm^2^) and 86.2% had C1-C2 instability (total >4.0 mm). Pearson correlation coefficient analysis showed a positive relationship between pupil diameter and intraocular pressure (*r* = 0.29, *p* < 0.01), while 20.7% had ocular hypertension.

**Discussion:**

Elevated optic nerve sheath diameter, elevated intraocular pressure, ocular dysautonomia, and succeeding eye symptoms may be pathophysiological effects of internal jugular vein compression and vagus nerve degeneration with underlying ligamentous cervical instability etiology.

## Introduction

An estimated 2.2 billion people suffered visual impairments worldwide in 2020 (1). Ligamentous cervical instability (LCI) and many eye conditions share the common characteristic of being progressive disorders necessitating vigilant monitoring and management to mitigate an ongoing impact on an individual’s health. There has been a reported 91.46% global increase in visual impairments from 1990 to 2019, with discernible unfavorable patterns in developed regions (2). With the ever-increasing usage of electronic devices comes myriad strains not only to the eye but to the cervical spine. “Computer vision syndrome” is associated with many ocular and musculoskeletal symptoms, including blurry vision, eye strain/fatigue, and head and neck symptoms (3). Recent evidence suggests that some eye symptoms may not be due to intraocular pathology stemming from the blue light emitted from the screen, as blue light-blocking spectacles do not significantly reduce symptoms in many people (4). Alternatively, symptoms may be due to destructive changes in the cervical spine from excessive poor posture, facedown/forward head posture (FD/FHP) that occurs with excessive screen time usage, also known as “tech neck” and “text neck” (5–7).

**Figure 1:**
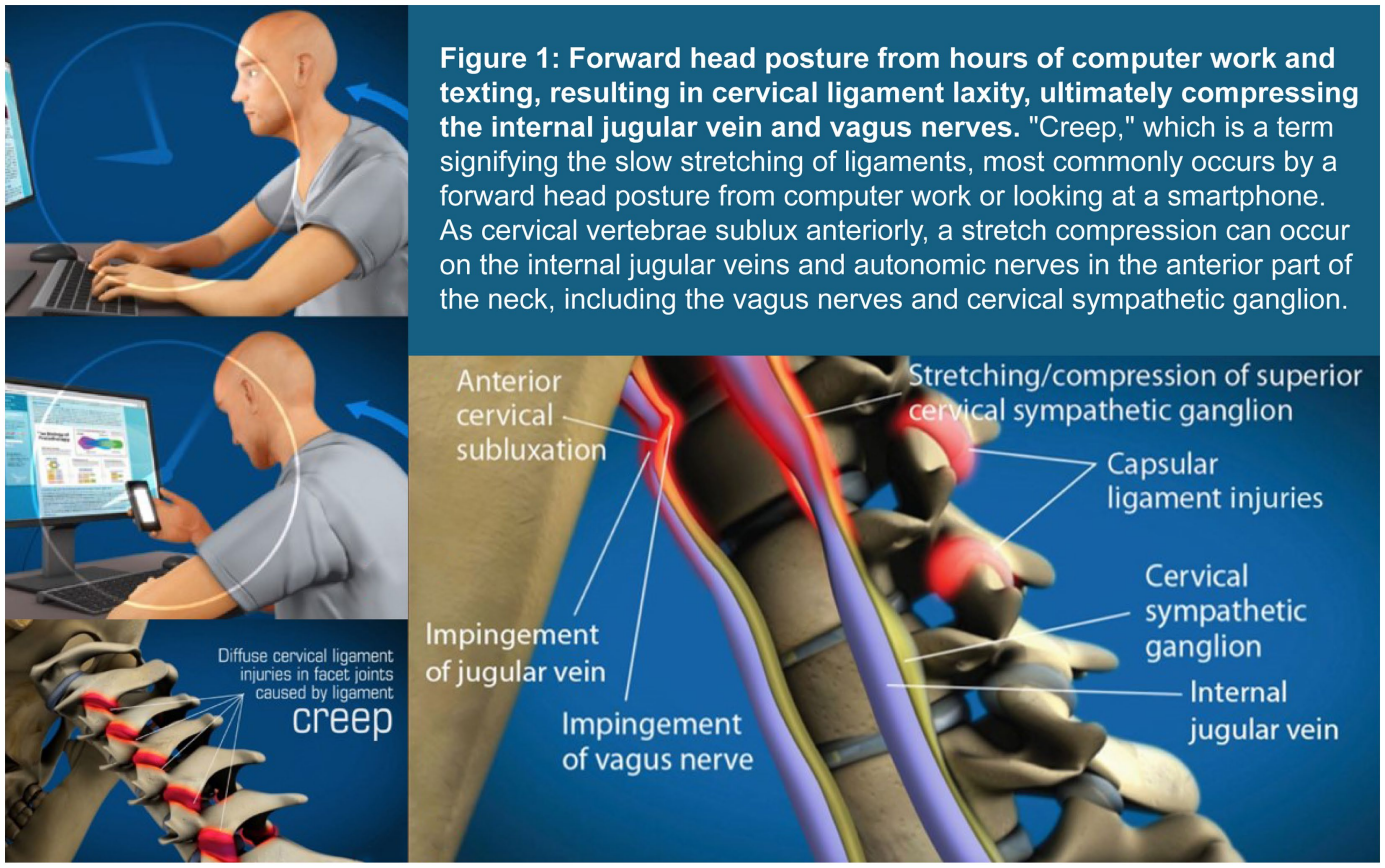
Forward head posture from hours of computer work and texting, resulting in cervical ligament laxity, ultimately compressing the internal jugular vein and vagus nerves. “Creep,” which is a term signifying the slow stretching of ligaments, most commonly occurs by a forward head posture from computer work or looking at a smartphone. As cervical vertebrae sublux anteriorly, a stretch compression can occur on the internal jugular veins and autonomic nerves in the anterior part of the neck, including the vagus nerves and cervical sympathetic ganglion.

Forward head posture is the most common cervical postural dysfunction and is associated with numerous eye symptoms, while text neck syndrome is also a postural manifestation stemming from inappropriate neck posture while looking at a computer or texting, and is associated with a host of musculoskeletal, psychosocial, emotional, and ocular symptoms (8, 9). The increasing 5–7+ hours on average that people, including children and adolescents, spend looking down at cell phones potentially causes many changes in the cervical spine, including elongation or stretching of the posterior ligament complex causing LCI, a breakdown of the cervical curve (cervical dysstructure), and overall changes in the sagittal plane, where the upper cervical spine shifts forward in relationship to the lower cervical spine (10, 11) (see Figure 1). Cervical structural changes can lead to compression of the carotid sheath and its contents, particularly at the level of the atlas (C1), causing a narrowing of the internal jugular veins (IJVs) and injury/degeneration of the vagus nerves. The former potentially leads to an increase in cerebrospinal fluid (CSF) pressure around the optic nerve head resulting in an elevated optic nerve sheath diameter (ONSD), and the latter contributes to ocular dysautonomia, with net effects of altered ocular hemodynamics causing elevated intraocular pressure (IOP), and resultant eye and visual symptoms (12, 13).

In this retrospective study, we hypothesized that a cohort of patients coming to an outpatient neck center with no known etiology for their pathology have objective test results that could explain not only their cervical structural pathology, but also their eye symptoms, including blurry vision, eye pain/pressure, eye tearing, light sensitivity, seeing flashes of light, and visual changes. To the best of our knowledge, this is the first study to demonstrate objective cervical structural and ocular test abnormalities together in a cohort of patients with no obvious etiology for their symptoms. The results support the hypotheses that an underlying LCI etiology may contribute to both musculoskeletal *and* eye symptoms in this patient population.

## Materials and methods

### Study population

The population included consecutive patients aged 20–50 years reporting to an outpatient neck center for care from January 1 to June 30, 2022. Informed consent was obtained from the subjects after explanation of the nature and possible consequences and the study was approved by the WIRB-Copernicus Group (WCG) Institutional Review Board (Study #1364545).

The inclusion criteria included patients who had at least 1 of 6 chronic eye symptoms (blurry vision, eye pain/pressure, eye tearing, light sensitivity, seeing flashes of light, and visual changes), but no obvious cause had previously been identified. Exclusion criteria were symptoms starting after a traumatic event (motor vehicle accident, sporting incident, surgery), or those that could be explained by a known diagnosis, such as ocular disease issues having required surgery or necessity of current usage of eye medication or eyeglasses.

We performed dynamic cervical structural, carotid sheath, and ocular tests, including measurements of IOP, pupil diameter, percent light constriction, and ONSD.

### Dynamic cervical structural testing

**Figure 2:**
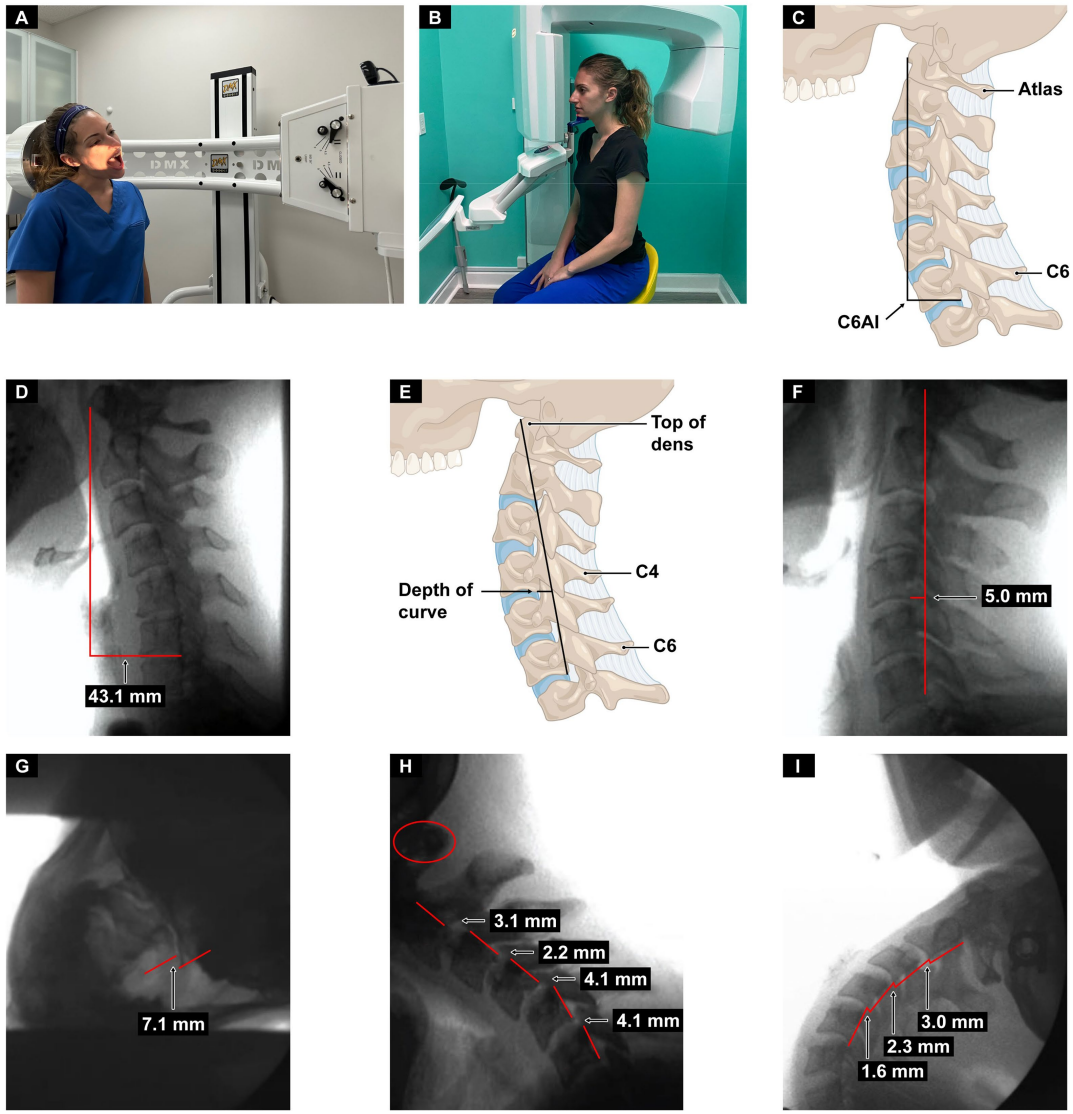
Upright digital motion (fluoroscopic) x-ray (DMX) and cone beam CT (CBCT) scan with structural measurements. **(A)** DMX positioning for open mouth lateral flexion. **(B)** CBCT setup. **(C)** Forward head (C6AI*) illustration. **(D)** C6AI measurement. **(E)** Depth of curve** illustration. **(F)** Depth of curve using DMX. **(G)** C1-C2 instability. **(H)** Flexion, lower cervical instability. **(I)** Extension, lower cervical instability. *C6AI = horizontal distance in the sagittal plane of the posterior inferior C6 vertebra to anterior atlas (optimal is <10 mm). **Depth of curve = horizontal distance in the sagittal plane from posterior inferior C4 vertebra to line drawn from posterior inferior C6 vertebra to top of dens (optimal is 7–17 mm).

All patients received an upright digital motion x-ray (DMX, videofluoroscopy) and upright cone beam computed tomography (CBCT) scan of the cervical spine, both done by a radiologic technologist. Measurements of ligamentous cervical instability in the lower cervical spine in flexion and extension, and in the upper cervical spine with open mouth views with lateral flexion, were taken. The C6AI measurements are defined as the horizontal distance from the posteroinferior border of the C6 vertebral body to a line drawn perpendicular from the anterior arch of the atlas (C1) in the sagittal view. Depth of curve (DOC) measurement is defined as the distance from the posteroinferior aspect of the C4 vertebra to a line drawn from the posteroinferior aspect of the C6 vertebral body to the peak of the dens of C2 (see Figure 2).

### Neck vitals analysis

**Figure 3:**
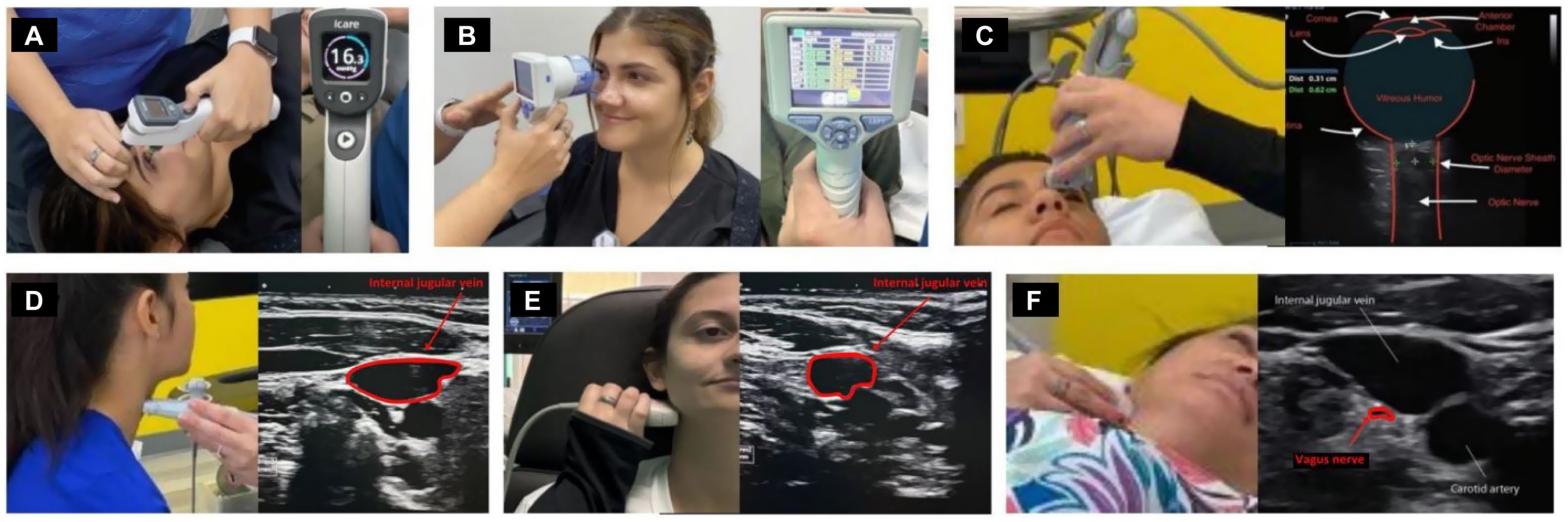
Neck vitals analysis. **(A)** Tonometry. **(B)** Pupillary light reflex. **(C)** Optic nerve sheath diameter. **(D)** Internal jugular vein (IJV) cross-sectional area (CSA) at C4-C5. **(E)** IJV CSA at C1. **(F)** Vagus nerve CSA.

All patients went through neck vitals analysis by a medical ultrasonographer, which included cervical and ocular ultrasounds and measurements of the vagus nerves’ cross-sectional area (CSA) mid-neck, IJV CSA in the supine position at C4-C5 and C1 (Canon Aplio a550 ultrasound with 7 MHz linear probe), pupil diameter and light reflex (NeurOptics NPi^®^-200 pupillometer), IOP (iCare ic200 tonometer), and ONSD (Canon ultrasound, ocular setting) (see Figure 3).

### Statistical analysis

The data were analyzed using RStudio 2024.04.2 + 764. Continuous variables were summarized using means and standard deviations, while categorical variables were summarized using counts and valid percentages (e.g., number of symptoms). Statistical significance was defined as *p* < 0.05 for all tests.

We began our data cleaning by removing all NA values from the dataset. Next, univariate outliers were identified and removed using z-scores exceeding ±3 (*n* = 17) to reduce the influence of extreme values. Analyses were conducted on both the full dataset (*n* = 203) and an outlier-removed dataset (*n* = 186) to assess the robustness of the findings. The sample size used in each test is reported accordingly.

Statistical assumptions for linear regression were then evaluated. Skew and kurtosis values of the scores fell within acceptable ranges (±3 and ±10, respectively). Histograms and Shapiro–Wilk tests were used to assess normality. While most variables were non-normal, Pearson’s correlation was used to assess the linear relationship between continuous variables. This decision was made for consistency across analyses and ease of interpretation, particularly given that most relationships appeared approximately linear and the sample size was sufficient to support the robustness of Pearson’s correlation to normality violations. Linearity was indicated by the correlation matrix and confirmed through visual inspection of scatterplots between continuous predictors and outcomes.

Next, paired t-tests were performed to compare the cross-sectional area of the internal jugular vein (IJV) across 3positional conditions: C1 supine, C4–C5 supine, and C1 supine on a Denneroll^®^ (a foam device that is placed under the neck in the supine position, intended to help restore the natural curve of the cervical spine). The results showed a statistically significant difference among the 3 conditions. Because the data were not normally distributed, however, a non-parametric Wilcoxon Signed-Rank Test was also used to confirm the results. Non-parametric alternatives to *t*-tests are more sensitive to violations of distributional assumptions and can provide more reliable inferences when comparing related samples with skewed data.

The analysis did not include covariates or multivariable adjustments because of the exploratory and observational nature of the study. Subgroup or interaction effects were not examined. The study did not employ a sampling strategy requiring weighting or design correction; all analyses were conducted using raw, complete-case data.

## Results

The demographics of the 203 patients who had at least 1 of 6 chronic eye symptoms is seen in Table 1, in which 94% had neck pain, 78% light sensitivity, 71% blurry vision, 67% eye pain/pressure, 67% vision changes, 36% seeing flashes of light, and 30% eye tearing.

**Table 1:** Demographics and symptom prevalence of 203 patients with at least 1 of 6 eye symptoms seen at an outpatient neck center during 2022.

Criteria	Number (%)
Demographics	
Average age	37.3
Males	101 (49.7)
Females	102 (50.3)
Number of symptoms	
1	25 (12.3)
2	36 (17.7)
3	39 (19.2)
4	41 (20.2)
5	43 (21.2)
6	19 (9.4)
Symptoms	
Neck pain	191 (94.1)
Light sensitivity	158 (77.8)
Blurred vision	145 (71.4)
Eye pain/pressure	136 (66.5)
Vision changes	135 (67.0)
Seeing flashes of light	72 (35.5)
Eye tearing	60 (29.6)

The mean and SDs of the dynamic cervical structural tests, as well as the total (bilateral) IJV and vagus nerve CSAs, pupillary diameters, light reflexes (percent constriction), ONSD, and IOP are summarized in Table 2. The mean C6AI, DOC, and total ligamentous cervical instabilities in the lower neck in flexion and extension, and the upper neck at the atlantoaxial facet joint of C1-C2 were 41.0 mm, 2.76 mm, 4.46 mm, 4.33 mm, and 7.29 mm, respectively. The mean total CSA of the bilateral vagus nerves mid-neck and IJVs in the supine position at C4-C5 and at C1 (without and with a Denneroll^®^) were 2.68 mm^2^, 127.5 mm^2^, 71.8 mm^2^, and 107.3 mm^2^, respectively. The mean total pupillary diameter, light reflex (percent constriction), ONSD, and IOP were 10.3 mm, 74.3%, 15.4 mm, and 35.9 mmHg, respectively.

**Table 2:** Neck vitals analysis summary^a^ (*n* = 203).

Criteria	Mean	SD
Age	37.32	8.68
C6AI^b^ (mm)	41.0	13.41
Depth of curve^c^ (mm)	2.76	3.58
Flexion instability (mm)	4.46	3.16
Extension instability (mm)	4.33	3.31
C1-C2 facet joint instability (mm)	7.29	3.25
Vagus nerve (CSA)^d^	2.68	0.80
IJV C4-C5 seated (CSA)	20.40	15.06
IJV C4-C5 supine (CSA)	127.52	81.87
IJV C1 seated (CSA)	20.66	16.12
IJV C1 supine (CSA)	71.75	42.50
IJV C1 Denneroll^®^ (CSA)	107.29	47.92
Pupil diameter total (mm)	10.27	1.52
Percent of light constriction	74.27	9.77
ONSD (mm)	15.37	1.74
Intraocular pressure (mmHg)	35.88	8.87

IJV = internal jugular vein, CSA = cross-sectional area, ONSD = optic nerve sheath diameter.

a The data were tabulated for bilateral measurements (except C6AI and depth of curve). Flexion and extension instability represent the total instabilities from C2–C7 seen.

b C6AI = horizontal distance in the sagittal plane of the posterior inferior C6 vertebra to anterior atlas (optimal is <10 mm).

c Depth of curve = horizontal distance in the sagittal plane from posterior inferior C4 vertebra to line drawn from posterior inferior C6 vertebra to top of dens (optimal is 7–17 mm).

d Vagus nerve CSA was taken at C4-C5 level, as there was so much compression at the atlas (C1) that it could not be viewed with ultrasound.

**Figure 4:**
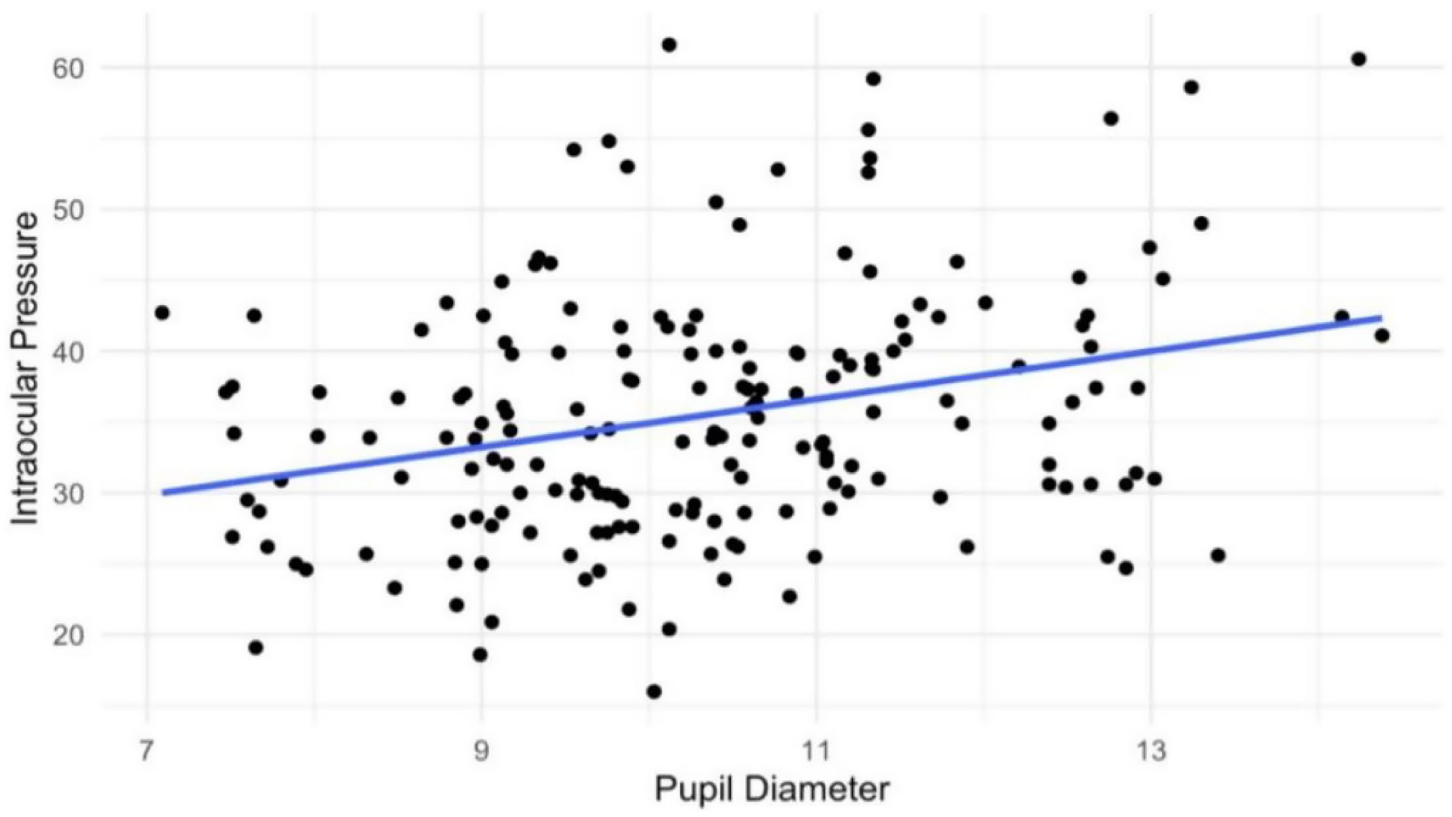
Scatter plot of pupil diameter vs. intraocular pressure. The Pearson correlation coefficient was positive at (*r* = +0.29 and *p* < 0.01).

After outliers were removed (using the z-score method with a threshold of 3 to ensure accuracy and reliability), a paired t-test conducted to compare the supine IJV CSA measurements at C4-C5 and C1 levels showed a significant difference between the 2 positions (*p* < 0.01). A Wilcoxon signed-rank test revealed a statistically significant difference comparing IJV CSAs with and without a cervical orthotic device (Denneroll^®^) at C1 (*p* < 0.05). A Pearson correlation coefficient analysis showed a positive relationship between the pupil diameter and intraocular pressure (*r* = 0.29, *p* < 0.01) (see Figure 4).

Table 3 describes the percentage of abnormal cervical structural and objective eye testing parameters. Over 90% of patients had compression of the IJVs at C1 (IJV CSA < 180 mm^2^), vagus nerve degeneration (vagus nerve CSA < 4.2 mm), excessive pupillary light reflex (>60%), and elevations of ONSD (>12.2 mm). Clinical ligamentous cervical instability at C1-C2 (total >4 mm) was seen in 86.2 and 20.7% had evidence of ocular hypertension (IOP > 42 mmHg).

**Table 3:** Summary of cervical structural and objective testing parameters that were abnormal in cohort of 203 patients with 1 of 6 eye symptoms.

Testing parameters^a^	Normal	% Abnormal (number)
Percentage of light constriction	<60%	94.6% (192)
Intraocular pressure	<42 mmHg	20.7% (42)
Optic nerve sheath diameter	<12.2 mm	98% (199)
Vagus nerve CSA^b^	>4.2 mm^2^	95.6% (194)
IJV CSA at C1, supine	>180 mm^2^	99.5% (202)
IJV CSA at C4-C5, supine	>180 mm^2^	77.8% (158)
C1-C2 facet joint instability^c^	<4 mm	86.2% (175)
Depth of curve^d^	7–17 mm	90% (182)
C6AI^e^	<10 mm	100% (202)

IJV = internal jugular vein, CSA = cross-sectional area.

a The data were accumulated for bilateral measurements.

b Vagus nerve CSA was taken at C4-C5 level, as there was so much compression at the atlas (C1) that it could not be viewed with ultrasound.

c C1-C2 facet joint—while some overhang of the C1-C2 facet joint on open mouth view is considered acceptable, people can be symptomatic even when the overhang is <2 mm on each side.

d Depth of curve = horizontal distance in the sagittal plane from posterior inferior C4 vertebra to line drawn from posterior inferior C6 vertebra to top of dens (optimal is 7–17 mm).

e C6AI = horizontal distance in the sagittal plane of the posterior inferior C6 vertebra to anterior atlas (optimal is <10 mm).

## Discussion

### Ligamentous cervical instability connection to eye symptoms

In this study, we found that a high percentage of people (86.8%, 203/234) coming to an outpatient neck center for care have at least 1 of 6 eye symptoms, 50.7% (103/203) suffering with 4 or more. As 94.1% (191/203) of the patients also experienced chronic neck pain, our hypothesis is that both the neck and the eye symptoms stem from a common etiology: LCI. Ligamentous spinal injury is characterized in medical literature by vertebral translation of more than 2 mm with various positions (14). The primary LCI found in our patient population was bilateral instability at C1-C2 with lateral flexion (86.2%, 175/203). This could explain not only the neck pain but also their eye symptoms, as 99.51% (202/203) had some degree of compression of the carotid sheath at the level of C1: total IJV CSA < 180 mm^2^.

While it is well known that visual symptoms are common in text neck and computer vision syndrome, the etiology remains debatable. This is the first study to document objective structural neck findings and neck vital analysis in a cohort of patients with chronic neck pain *and* eye symptoms without a known cause. The emphasis in the scientific literature has been focused on digital eye strain from such things as reduced blink rate, poor accommodation (focusing fatigue), blue light exposure, and convergence stress (binocular dysfunction) (5–7). Our study suggests with objective evidence that poor ergonomics via the FD/FH lifestyle may be the primary culprit.

Many studies have already suggested bidirectional links between neck pain and eye symptoms, often emphasizing a postural contributing factor, for example with call center employees and screen time of university students (15, 16). A growing body of evidence further demonstrates a connection between cervical spondylosis and visual dysfunction, though mechanisms remain controversial and unclear (17). As such, there is limited literature available for direct comparison. Patients presenting to the clinic in which the study took place often report after seeing many other specialists, including neurologists and ophthalmologists, and after undergoing traditional diagnostic testing without a clear explanation or diagnosis. The data presented here aim to guide further exploration of neck and eye associations, to potentially uncover what could be causing musculoskeletal complaints *and* eye symptoms in a select subset of patients whose symptoms have gone otherwise unresolved.

As our patient population had no known one-event trauma or previously identified structural eye issue, and the symptoms occurred insidiously, the dynamic cervical structural changes are likely related to a “normal” but excessive amount of time spent in poor posture hunched over a cell phone or looking at a computer screen causing the slow stretching, and thus elongating, of the cervical ligaments, a process known as cervical creep (18, 19). One of the primary responses of the body to ligament injuries is muscular tension through the ligamento-muscular reflex (20). LCI is characterized by posterior muscular tension (giving pain) with certain movements to limit anterior translation of the cervical vertebrae as a protection mechanism for the neurovascular structures in the neck, including the carotid sheath contents (21). Also giving credence to LCI etiology is our relatively young patient population (average age 37.3 years), making it doubtful the various symptoms are due to degenerative conditions or the natural effects of aging. LCI represents an unexplored explanation for the ever-growing phenomenon of text neck syndrome and the accompanying musculoskeletal pain and eye symptoms (10).

LCI was evaluated by DMX (videofluoroscopic) examination, a known valid measure for cervical spine ligament injury, which allows for a continuous and detailed examination of cervical spinal movement and unrestricted assessment of C0-C7 motion in multiple dimensions, including the sagittal, rotational, and frontal planes (22). The DMX studies show the functional integrity of the ligaments in the cervical spine, specifically the anterior and posterior longitudinal, supraspinous, interspinous, ligamentum flavum, transverse, alar, and facet capsular ligaments. LCI is defined by the degree of overhang or subluxation (excessive motion) of adjacent cervical vertebrae. In our patient population, upper cervical (C1-C2) instability was much greater than that found in the lower cervical regions (C2-C7): 7.29 mm total lateral flexion at C1-C2 vs. 4.46 mm in flexion and 4.33 mm in extension in the lower cervical.

**Figure 5:**
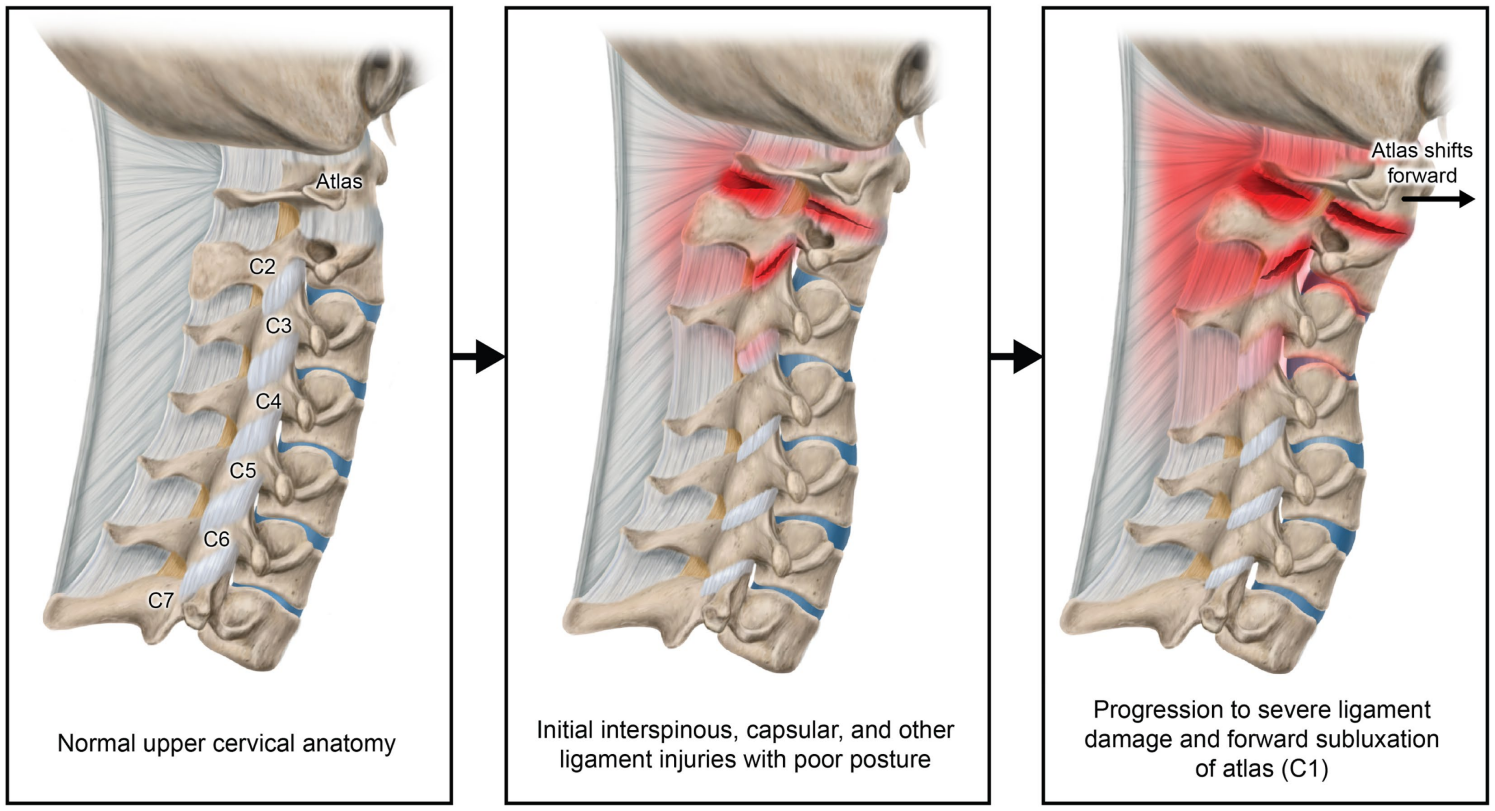
Lateral view of cervical spine demonstrating strain on upper cervical ligaments with forward head posture.

**Figure 6:**
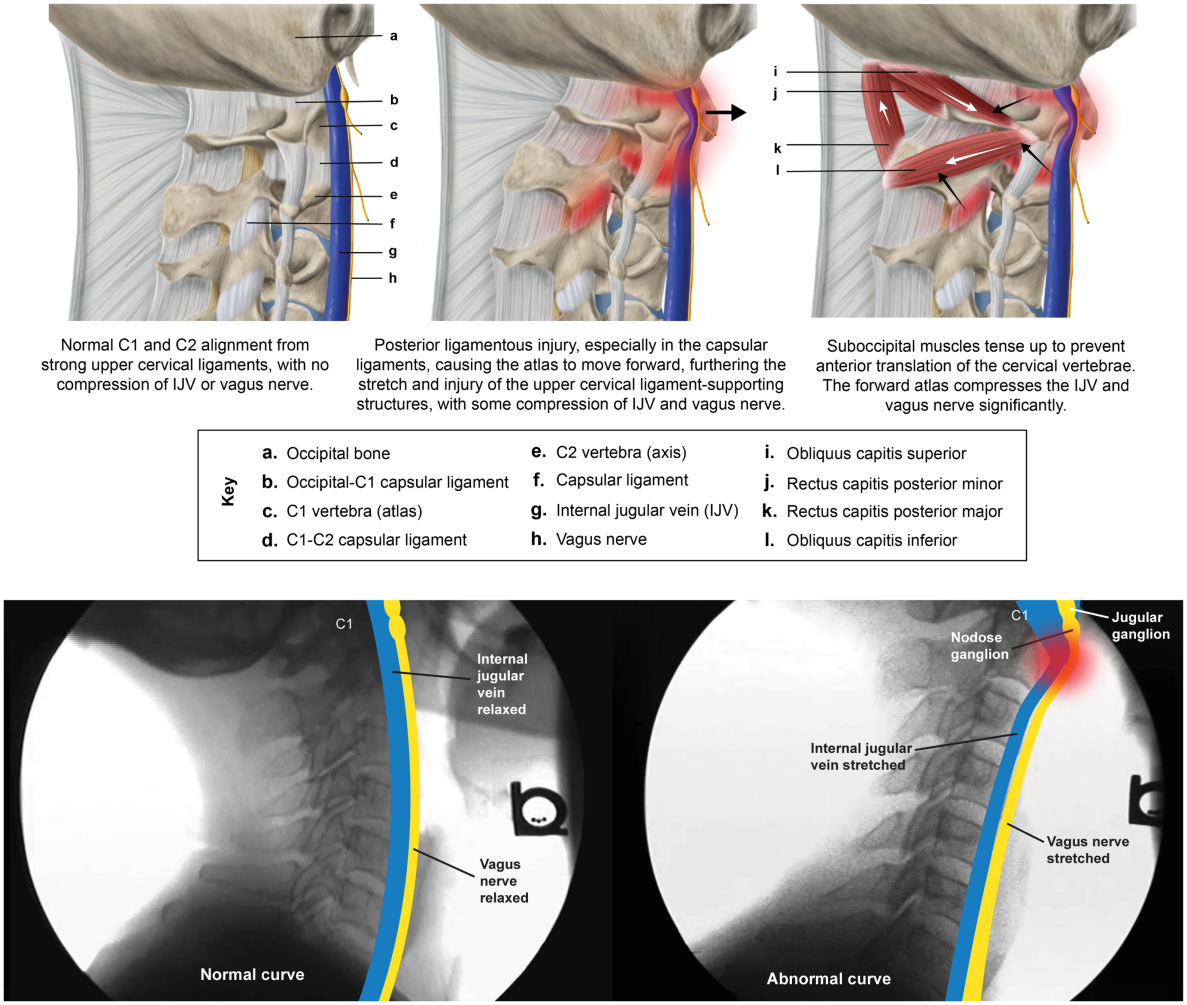
Pathology caused by ligamentous upper cervical instability in illustration and x-ray. Ligamentous upper cervical instability stimulates the ligamento-muscular reflex to limit atlas motion, resulting in craniocervical pain and stiffness, whereas the forward atlas compresses the carotid sheath, leading to internal jugular vein compression and vagus nerve degeneration and dysfunction.

The static cervical structure was evaluated by upright CBCT (sagittal x-ray used if C6 vertebra not seen with CBCT) measuring the DOC and C6AI. In our patient population, the cervical lordosis depth mean was decreased at 2.76 mm, normal cervical lordosis depth being between 7–17 mm as evaluated by the Borden method (23, 24). The average amount of forward displacement of the atlas compared to the lower cervical vertebra (C6) was 41 mm (1.6 inches) in the sagittal plane (ideal <10 mm) (25–27). When the ligamentous support of the neck becomes dysfunctional from FD/FHP, it causes a breakdown of the normal cervical lordotic curve that we term cervical dysstructure, documented by decreased DOC and C6AI (28). The FD/FHP involves forward protrusion of the head in relation to the trunk in the sagittal plane, causing the lower neck (C2-C7) to be in constant flexion, which necessitates the upper cervical (C0-C2) to go into extension to maintain a stable horizontal gaze, putting additional strain on the upper cervical ligaments (8, 29–31) (Figure 5). The larger the neck flexion angle, the greater the forces on the posterior soft tissue structures in the neck, which triples at 40^°^ (text neck) compared to neutral, making cervical instability more probable (31–34). The combination of FD/FHP (documented by C6AI) and LCI (specifically, C1-C2 capsular joint instability), along with resultant cervical dysstructure, is a setup for compression of the carotid sheath at the level of the atlas (C1), causing various eye symptoms to arise due to venous outflow obstruction and ocular dysautonomia (see Figure 6).

### IJV compression, CSF pressure, and ONSD

**Figure 7:**
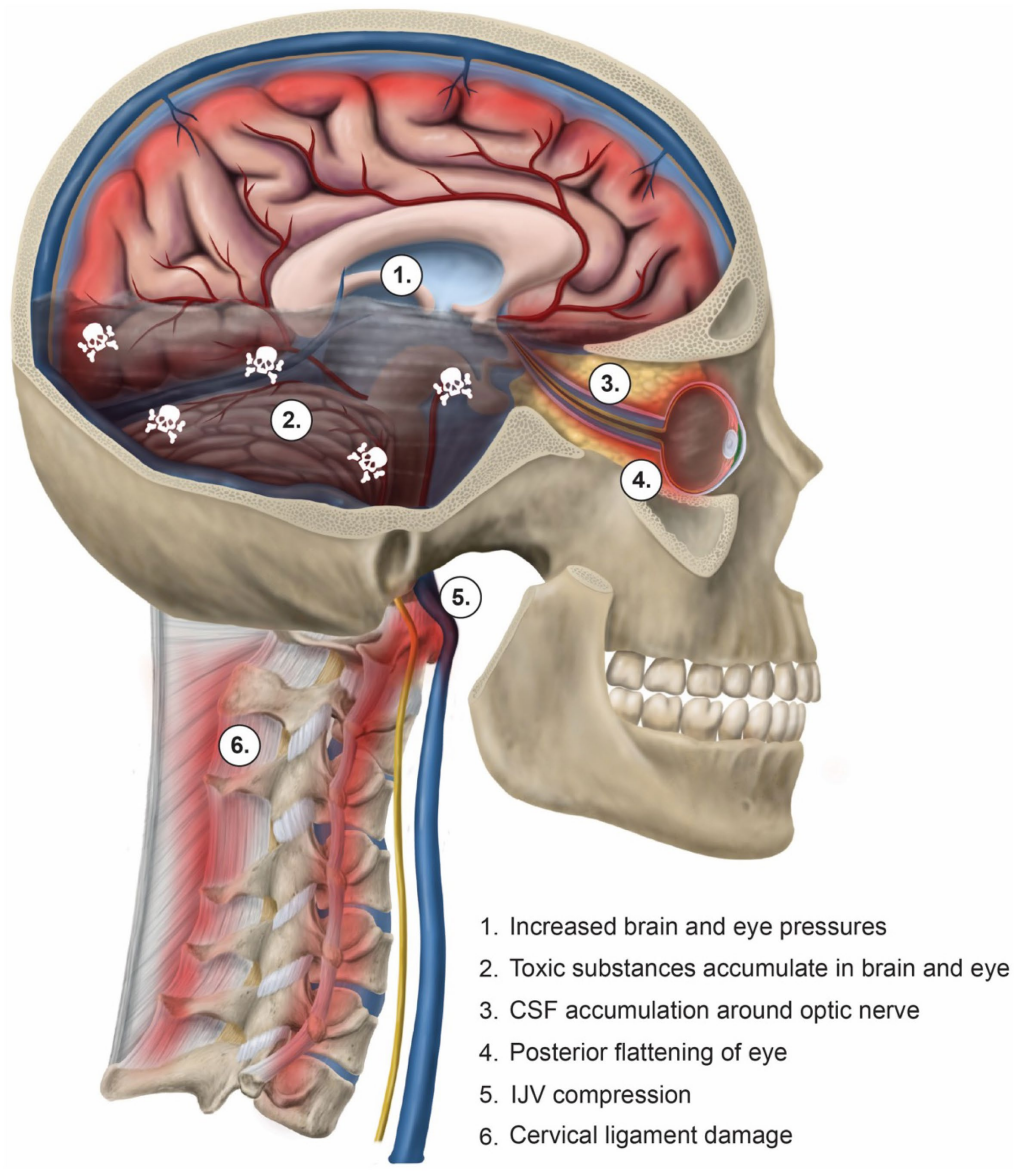
The toxic degeneration of the brain and eye from internal jugular vein (IJV) compression. A breakdown of the cervical curve (dysstructure) from ligamentous cervical instability results in compression of the IJV, which hampers the removal of metabolic waste products form the brain and eye. Together with the resultant high brain and eye pressures, this leads to toxic degeneration of both organs.

Consequences of IJV compression affect not only the eye, but also the brain (35). A reduction in venous outflow in the supine position can increase cerebral volume and intracranial pressure, resulting in an increase in CSF, cerebral venous sinus, and episcleral venous pressures, potentially causing increased IOP and accumulation of CSF around the optic nerve (elevated ONSD) (36–41). The net effect of this compression can be inadequate metabolic waste product removal from both the brain and the eye (42, 43) (see Figure 7). In this patient population, compression of the carotid sheath in the supine position was documented primarily by decreased IJV CSA at the level of the atlas (C1). Fully 99% of the patients had evidence of bilateral total IJV compression (<180 mm^2^) and 98% of the patients had elevated bilateral total ONSD (>12.2 mm), with the mean bilateral ONSD being 15.37 mm.

Measurements of IJV CSA at C1 were statistically smaller than at the C4-C5 level (*p* < 0.01), with a mean difference of 53.3 mm^2^. Remarkably, IJV CSA at C1 was statistically greater when using a cervical lordotic device, the Denneroll^®^, to enhance the cervical lordotic curve (mean 105.23 mm with Denneroll^®^ vs. 69.47 mm without, *n* = 186, *p* < 0.05), implicating loss of cervical lordosis (decreased DOC) as a potential etiology of carotid sheath compression. The Denneroll^®^ is a foam device that is placed under the neck in the supine position, intended to help restore the natural curve of the cervical spine.

**Figure 8:**
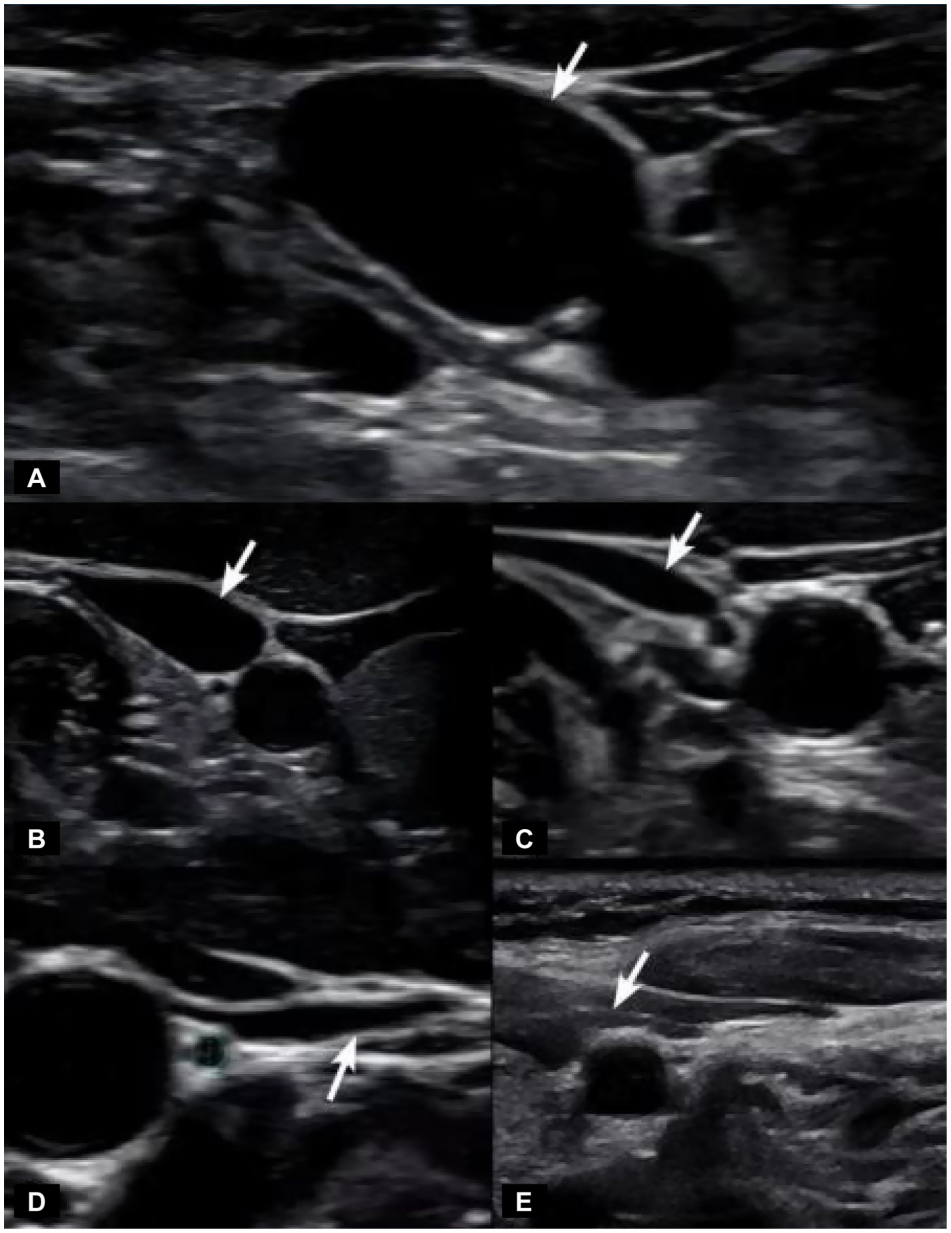
Degrees of internal jugular vein compression as seen on ultrasound examination of the neck. **(A)** Normal “open” internal jugular vein. **(B)** Slightly compressed. **(C)** Moderately compressed. **(D)** Severely compressed. **(E)** Completely closed. It is internal jugular vein compression (arrows), especially with upright posture and neck motions, that leads to intracranial hypertension (increased brain pressure).

IJV CSA is easily measured under B-mode ultrasound (see Figure 8). Unilateral normal IJV CSA in the supine position is >90–100 mm^2^ (44–46). As IJV CSA is typically measured in the mid-cervical region, our study documents that measuring it at the level of the atlas was a more sensitive test for IJV compression when evaluating with ultrasound (42, 47). Not only does LCI promote excessive motion of the atlas, but as the cervical curve breaks down, the atlas shifts forward in 3-D space. In our patient population, the documented LCI, decreased DOC, and increased C6AI likely account for the bilateral IJV compression at C1.

Jugular venous outflow insufficiency is one major cause of intracranial hypertension and is associated with many ophthalmological manifestations, including visual loss, blurred vision, eye pain, and photophobia, and also neck pain and head pressure (48–50). Elevated CSF pressure due to IJV compression is one mechanism that could explain many of those associated conditions. When CSF pressure rises and accumulates in this area at the back of the eye where the optic nerve enters the globe, it can cause posterior globe flattening or optic nerve tortuosity, which can be seen on MRI imaging, and is known to be correlated with intracranial hypertension and increased IOP (51–54). Any type of compressive pressure along the optic nerve can negatively affect vision.

**Figure 9:**
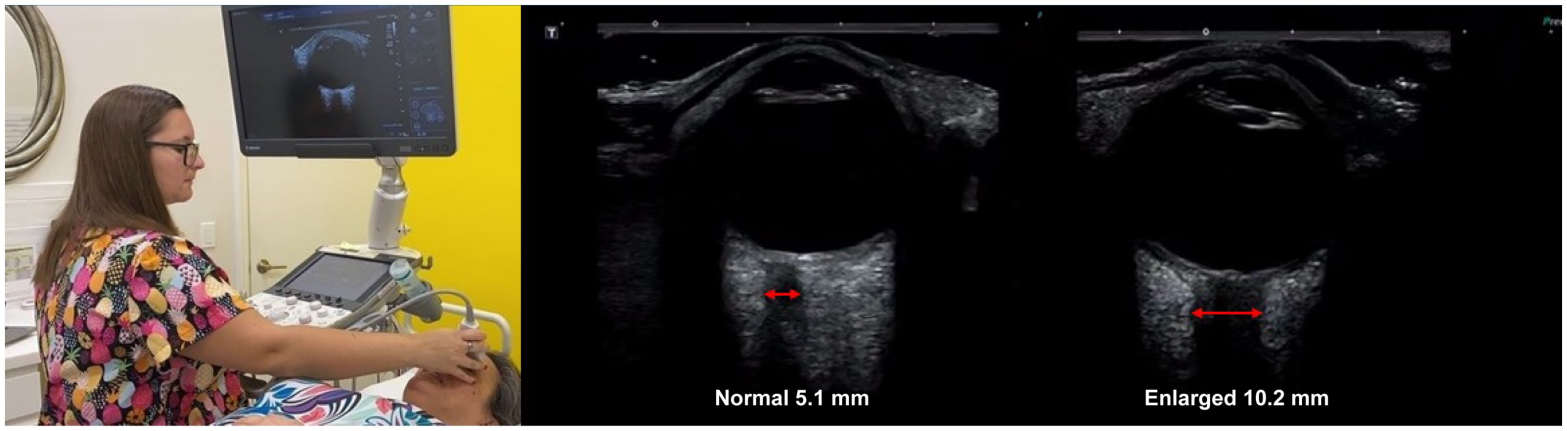
Ultrasound of eye to measure optic nerve sheath diameter, which is used as a marker for intracranial hypertension (increased brain pressure).

Ultrasound ONSD measurement is a valid noninvasive imaging technique to detect and monitor intracranial hypertension, as the optic nerve is surrounded by CSF through the subarachnoid space (55–58). The optic nerve emerges from the posterior part of the globe and appears as a hypoechoic linear structure with a hyperechoic border (nerve sheath). The outer rim should be included in optic nerve sheath measurements and should be measured 3 mm behind the posterior rim of the globe (where standard measurements are made.) Measurements in healthy adults are generally between 4.9–5.3 mm (59). Emergency rooms and others consistently use an ONSD measurement greater than 6.0 mm to diagnose intracranial hypertension, though some use an even lower cut-off (60–64) (see Figure 9).

Elevated ONSD could be due to compression of the IJV, disturbing cerebral drainage and increasing CSF and intracranial pressures. The increased pressure surrounding the optic nerve can affect the transmission of the eye image from the retina to the occipital lobe and account for the high percentage of people in our patient population complaining of various visual distortions, including blurry vision, vision changes, and even seeing flashes of light (37, 65). Self-reported vision changes and flashes of light are often obscure but commonly reported symptoms. Intermittent vision changes, including flashes of light, have been associated with posterior vitreous detachment and migraine with aura (66–68). One possible explanation in our patient cohort is that elevated ICP and IOP could be contributing to disrupted visual processes, as they both influence the retina and optic nerve (69, 70).

While the validity of pooled sensitivity and specificity of ONSD measurement by ultrasonography were 84% (95% CI, 76–89%) and 83% (95% CI, 73–90%) in documenting intracranial hypertension, ONSD measurements are operator dependent based on the experience of the ultrasonographer. Future studies using serial CT or MRI venography could help verify the compression of the IJVs primary at the level of the atlas compared to the mid-cervical region as found in this study (71, 72).

### Ligamentous cervical etiology for ocular dysautonomia

**Figure 10:**
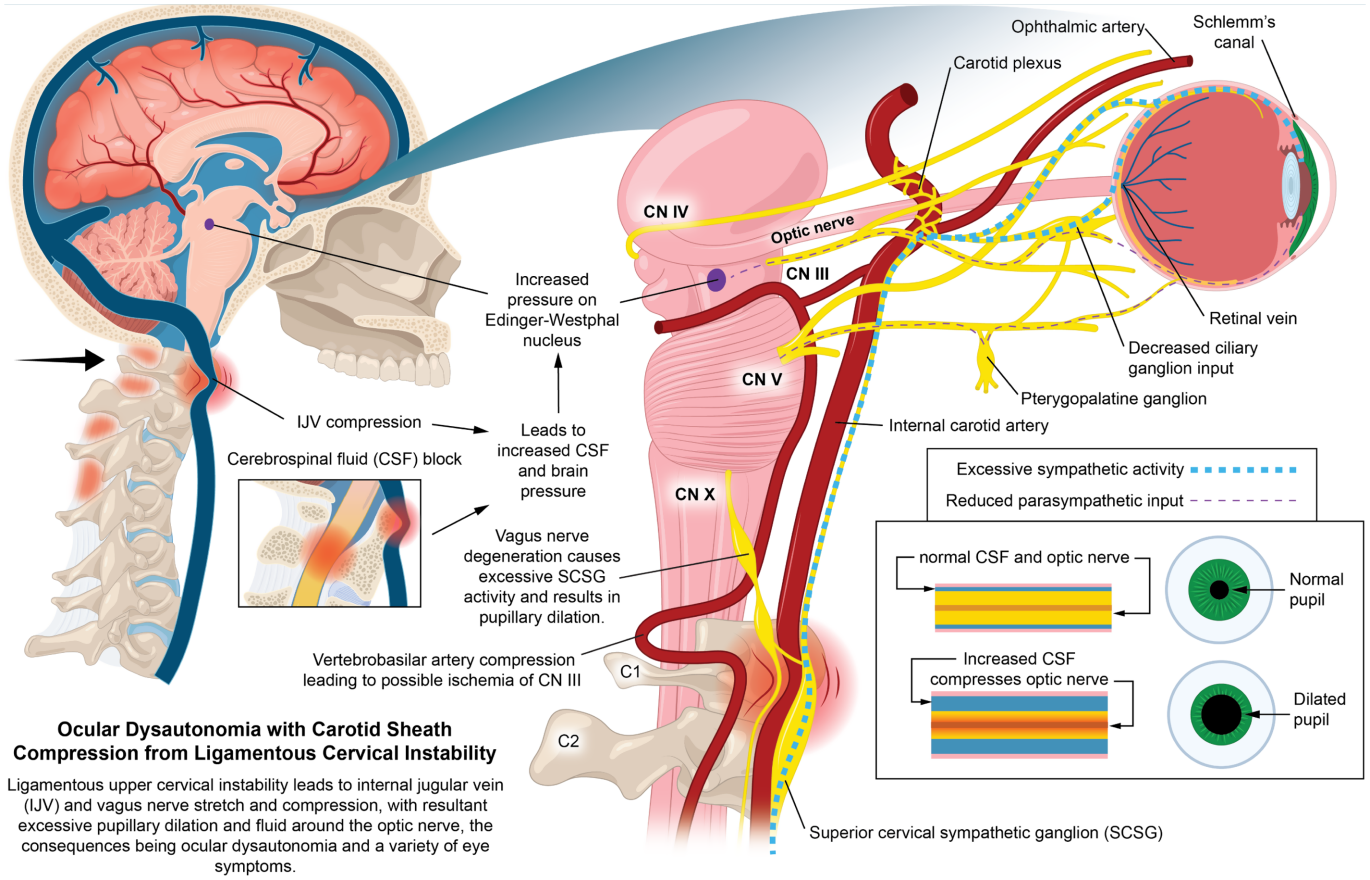
Ocular dysautonomia with carotid sheath compression from ligamentous cervical instability. Ligamentous upper cervical instability leads to internal jugular vein (IJV) and vagus nerve stretch and compression, with resultant excessive pupillary dilation and fluid around the optic nerve, the result being ocular dysautonomia and a variety of eye symptoms.

Mechanisms by which LCI can affect the autonomic nervous system control of the eye include: cranial nerve dysfunction by increasing CSF pressure around the nerves, elevated IOP by inhibiting cerebral venous drainage, cervicovagopathy (vagus nerve degeneration from cervical pathology), sympathetic dominance by effects on the superior cervical sympathetic ganglion (SCSG) and sympathetic nerves, pressure on vagal ganglia or the Edinger-Westphal midbrain nucleus, and trigeminal nerve dysfunction secondary to effects on the trigeminocervical nucleus (73–75) (see Figure 10).

Ocular dysautonomia was documented in this study, with 91% of eyes having an excessive percent change in pupil diameter ([max.–min.]/size as max. %) (76). The mean total percent change was 74.3% (normal limit ≥30–60%) (77–79). Proper vision requires an appropriate amount of light entering the eye, which is determined by influences of the sympathetic nervous system/ parasympathetic nervous system pathways to the pupil. Some 77.8% (158/203) of this patient population suffered with light sensitivity that we suspect to be caused by excessive pupil diameter, which averaged 5.1 mm, or 10.27 mm total. Most common pupil diameters in healthy individuals are between 2–4 mm (80, 81).

Quantitative pupillary light reflex is widely used for the detection of imbalanced autonomic nervous system (82, 83). Traditionally, abnormal pupillary response is thought to occur because of damage to the optic or oculomotor nerves, brainstem abnormalities, or medications (84). In our patient population, the sympathetic dominance causing excessive pupillary constriction percentages in response to light and slightly elevated baseline pupil diameters is likely in part from the high prevalence of patients’ having vagus nerve degeneration (95.6%, 194/203), considering the vagus nerves carry 75%–80% of the parasympathetic nervous system fibers and their direct interconnectedness with the SCSG (decreased vagus nerve signaling to SCSG would cause increased SCSG sympathetic outflow to eye) (85, 86).

Sympathetic overdrive has been shown to concur with sympathetic pupillary responses in migraine and cluster headaches (87, 88). Vagus nerve degeneration may contribute to sympathetic dominance without impairing the pupillary light reflex, even though the pupillary light response is mediated by parasympathetic fibers in the Edinger-Westphal (EW) nucleus, as the vagus nerve and EW nucleus can indirectly interact. This mediation could allow normal pupillary response to be preserved, but with resultant increased baseline pupil diameter and increased percentage (percent change) constriction to light. A higher constriction percentage is to be expected if the pupillary response is intact and the baseline pupil diameters are on the high end of the normal range before the stimulus (79, 89). Further studies are encouraged to investigate potential relationships between vagus nerve degeneration and pupillary insights.

**Figure 11:**
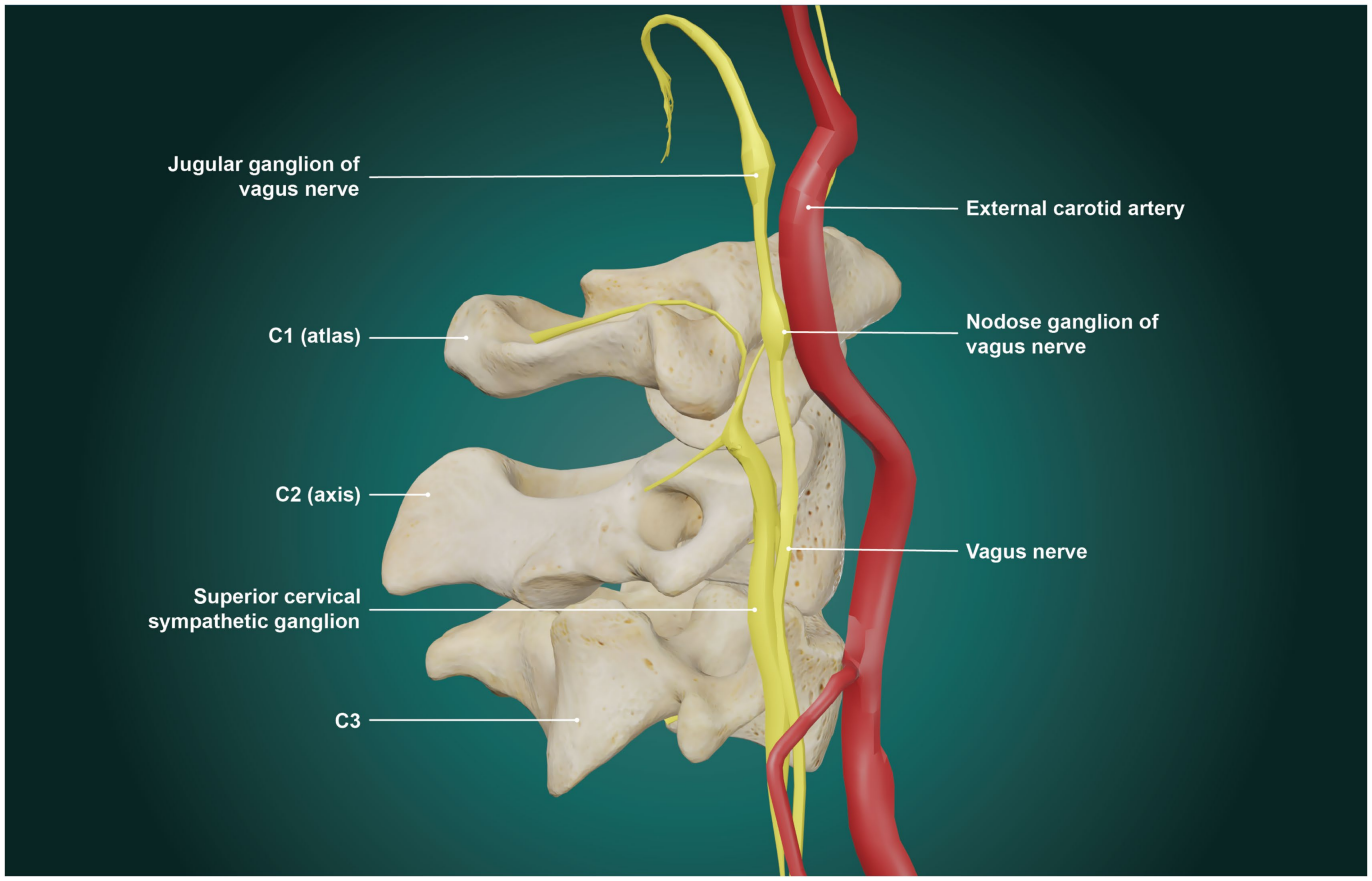
Relationship of superior cervical sympathetic ganglion (SCSG) to vagus nerve and internal carotid artery (ICA). The SCSG typically lies lateral to the vagus nerve and posterior to the ICA.

The mean bilateral vagus nerve CSA of this patient population was 2.68 mm (normal limit ≥4.2 mm). The vagus nerve’s influence on the eye is primarily accentuated antagonism, as it modulates the presence of background sympathetic stimulation (90). The SCSG is a primary sympathetic nerve supply to the eye, typically located right in front of the C2 and C3 transverse processes, medial to the vagus trunk, anterior to the longus capitis muscle, and posterior to the internal carotid artery (see Figure 11). The SCSG influences pupil dilation and blood flow to the eye, and has been implicated in many conditions and symptoms that include elevations of intraocular pressure, glaucoma, photophobia, and macular degeneration (91–95). The nodose ganglion (inferior ganglion of the vagus nerve) sits directly anterior to the atlas. The location of both, traversing the anterior neck, make them easily compressed or stretched by LCI and cervical dysstructure. When sympathetic activity is too great, as expected with vagus nerve degeneration, the most common symptom produced is light sensitivity (photophobia), which was seen in 77.8% of this patient population (96). Vagus nerve degeneration may also explain eye tearing, seen in 30% of this patient population, being a possible effect of parasympathetic dysfunction affecting the control of the lacrimal and Meibomian glands (97, 98). Regarding the novel finding of vagus nerve degeneration in this patient population and its role in ocular dysautonomia, more direct evidence in future studies will be needed to substantiate this claim.

### Ligamentous cervical etiology for ocular hypertension

**Figure 12:**
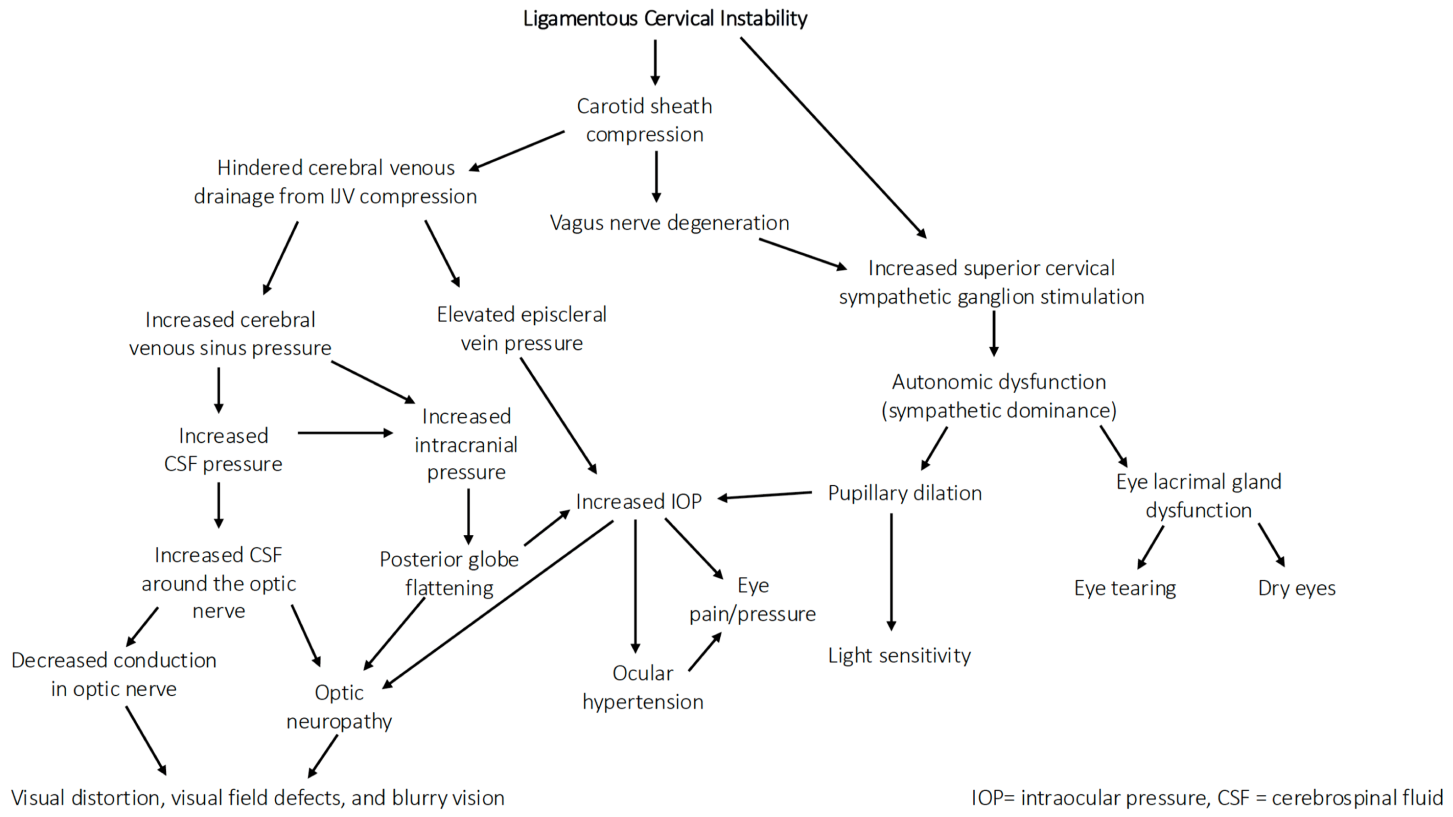
Ligamentous cervical instability and the potential long-term consequences to the eye.

There are many mechanisms by which ligamentous cervical instability could lead to ocular pathology, including ocular hypertension (see Figure 12). While normal eye pressures are between 10–15 mmHg, ocular hypertension is defined as IOP > 21 mmHg (99). In our patient population, intraocular pressure showed a positive correlation with pupil diameter (*r* = 0.29, *p* < 0.01). The mean IOP measurement was 17.2 mmHg, and 20.7% met diagnostic criteria for ocular hypertension. A 2023 study done in Bangladesh, however, shows that up to 9% of the general population over 40 years of age meet the criteria for ocular hypertension (100).

**Figure 13:**
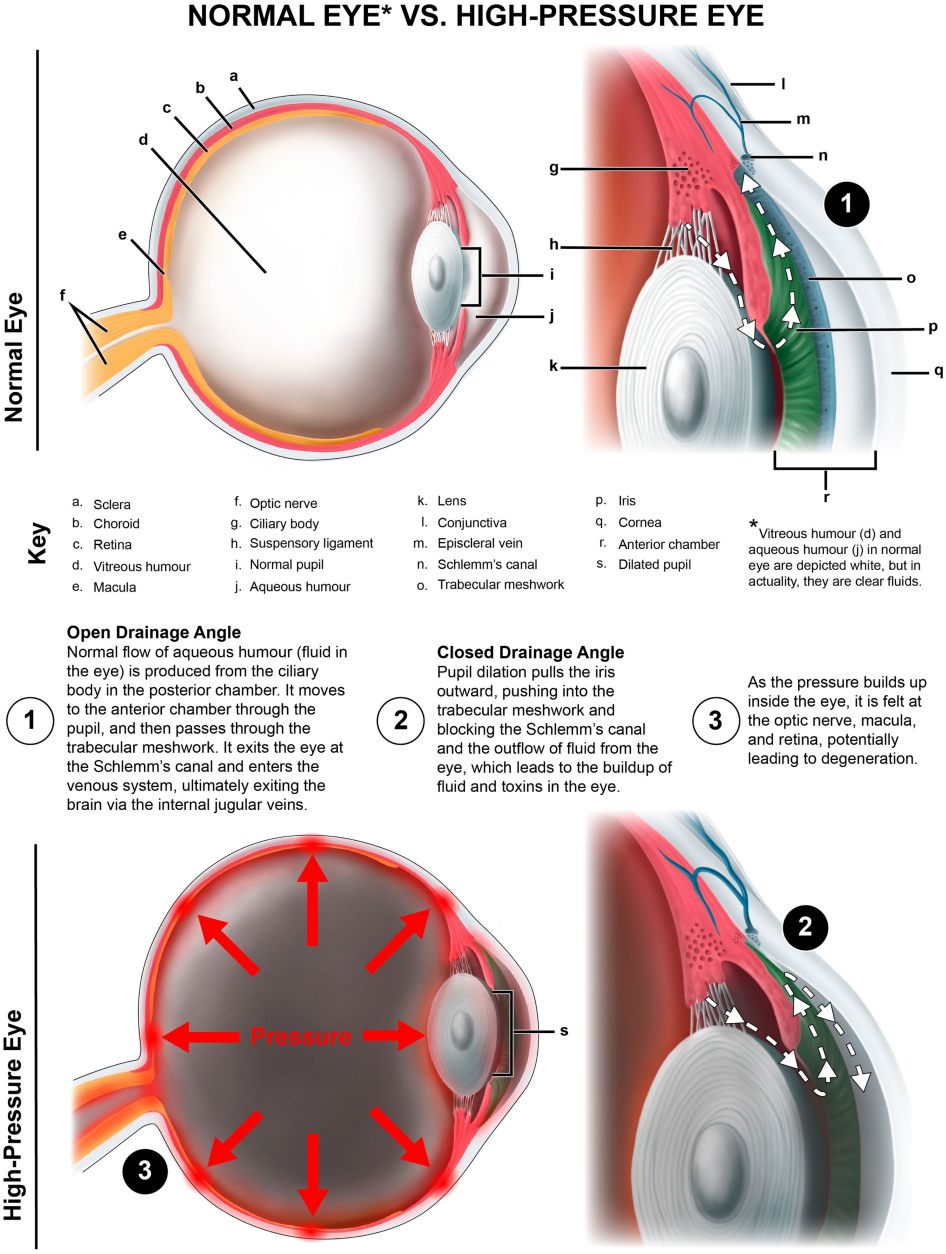
Normal eye vs. high-pressure eye.

In this study, patients with previously existing conditions that would warrant the use of a pharmacological agent were excluded. Some cases of elevated IOP in this patient population may be due to baseline low-level pupil dilation, a known trigger for increasing IOP (101, 102). Pupil size is influenced by multiple factors, including the autonomic nervous system, ambient light, and pharmacological agents. Not as commonly considered is that the pupil dilation may be brought on by vagus nerve degeneration or IJV compression, the former causing dilated pupils via sympathetic dominance and the latter causing dilated pupils due to a buildup of pressure inside the eye, either from an upstream effect of elevated pressure in the episcleral veins, or from pressure on the back of the orbit due to an accumulation of CSF (34, 103, 104). Pupil dilation pulls the iris outward, pushing into the trabecular meshwork and blocking Schlemm’s canal, where a majority of the aqueous humor should flow to exit the eye to maintain ocular homeostasis. Future studies should consider gonioscopy findings to assess the status of the anterior chamber angle and evaluate angle closure as a cause of elevated IOP. Other causes of increased IOP include autonomic dysfunction, excess aqueous humor production, and resistance to aqueous humor drainage through related pathways (105). Elevated IOP can lead to eye pain/pressure, ocular hypertension, and associated symptoms such as blurry vision and visual changes, potentially progressing to ocular disease (106, 107) (see Figure 13).

### Cervical structural treatment for eye conditions?

**Figure 14:**
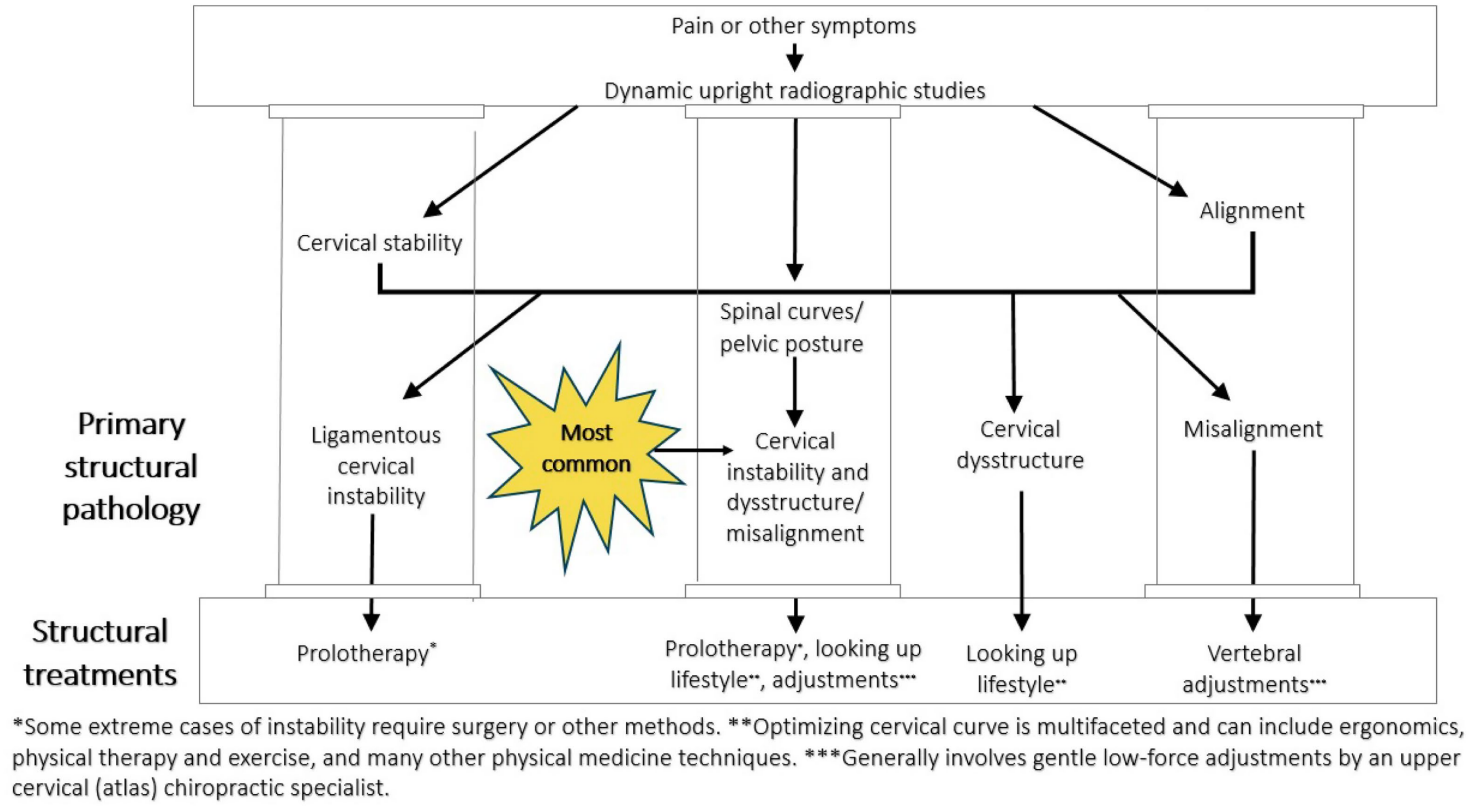
Cervical treatment recommendations based on dynamic upright radiographic studies. Patients often have a combination of ligamentous cervical instability, cervical dysstructure (breakdown of cervical curve), and misalignments, the 3 pillars of cervical structural health.

Overall, we found that this study supports the hypothesis that some eye symptoms with otherwise unknown etiology may be associated with ligamentous cervical instability due to the net effects of IJV compression and vagus nerve degeneration. If eye symptoms are related to changes in the cervical curve that occur because of a FD/FHP lifestyle by the slow stretching of the cervical ligaments, then resolution of such symptoms might occur with therapies such as physical therapy, therapeutic exercises, postural ergonomic changes, gentle chiropractic or osteopathic adjustments, and therapies that assist in restoration of the cervical curve and its stabilization by tightening of the ligaments with Prolotherapy (18, 108, 109). The goal of the therapies is relieving vascular or neurologic structural abnormalities affecting the eye (see Figure 14).

To our knowledge, this is the first study to examine objective cervical structural and ocular clinical data in a cohort of patients presenting with various eye symptoms. We believe that this exploratory analysis offers meaningful insights by highlighting clinical patterns which may be underrecognized in conventional diagnostic settings. We aimed to present associations that could connect emerging interdisciplinary observations, not to draw definitive mechanistic conclusions. Future research should incorporate formal ophthalmic testing and cervical spine assessments to test these hypotheses. We recommend the replication of the discussed diagnostics, including cervical structural and neck vitals analysis, in future studies to confirm cervical spine pathologies, especially neurovascular compression at C1. The goal of exploring potential mechanisms and associations is to utilize dynamic structural medicine treatment protocols to potentially improve outcomes of patients with otherwise elusive etiology of their eye symptoms.

While our patient population had previously seen eye specialists for standard evaluations without diagnosis or successful treatment, it is possible that conditions associated with normal aging processes of the eye, such as presbyopia, still exist and could be corrected using eyeglasses, which could contribute to the development of poor posture and resultant neck pain (110, 111). In such cases, eye symptoms and visual strain are likely to contribute to poor posture while using electronic devices, especially for prolonged periods, putting strain on the cervical spine, which leads to LCI, resulting in a vicious cycle until both the eye problems and cervical neck structure are addressed (112–114). To date, there are no data that show normal aging of the eye relates to abnormal dynamic pupillometry analysis or elevation of ONSD as we documented in this unique subset of patients, although patients with hyperopia may experience earlier-onset symptoms of presbyopia due to their increased dependence on accommodation (15). The multifaceted relationship that exists between the cervical spine and the eye highlights the necessity for future research and comprehensive, interdisciplinary assessment, as well as development of preventative approaches, including postural guidance.

### Limitations

The study was limited by the study group not having a comprehensive ophthalmologic examination of the eye, including the optic nerve head and specific retinal examinations such as optical coherence tomography or visual acuity testing. In future studies, formal gonioscopy examinations to access the anterior chamber angle should also be included, which would be helpful in interpreting IOP evaluations.

As there was not a control group with normal, stable cervical lordotic anatomy to compare symptom frequencies and diagnostic testing results with our patient population, we could not do a comparison study with a control. Future studies should further investigate the potential relationships between the neck and the eye in a controlled setting where objective test findings can be compared between normal lordotic curves without LCI and those with LCI and cervical dysstructure. We also did not have a control group without eye symptoms to compare if there was any difference between the groups in regard to the duration of neck or eye symptoms and the various objective tests that were done. Without any control groups, we cannot determine whether the observed results are specific to this patient population or could also possibly present in the general population. This study did not compute how much time each participant spent in poor posture from electronic device use preceding eye symptoms, therefore FD/FHP as a preceding event is speculative. The study excluded anyone with previously diagnosed or identifiable conditions and anyone over the age of 50 to reduce bias of natural aging processes, but the study is limited by any potentially overlooked conditions caused by natural aging. Future studies might also consider the timeliness of corrective eyeglasses for looking at and reading on digital screens at short distances to prevent visual strain and consequently FD/FHP, leading to LCI in cases where the symptoms are corrected by use of eyeglasses.

The study design demonstrates associations between ligamentous cervical instability, objective test results, and ocular symptoms, but does not establish causality. The observed correlations between ligamentous cervical instability and ocular findings could be coincidental, or due to confounding factors such as systemic conditions that could simultaneously affect both the cervical spine and the eyes. We present this preliminary data with plans to carry out more rigorous studies that will further evaluate the discussed hypotheses, and to prompt further investigation. Since these data were from initial presentations to the clinic, future longitudinal studies that show improvements of neck structure by various therapies and correlating them to symptom and diagnostic testing result improvements, would help confirm the connection between neck structure and eye symptoms and pathology.

## Conclusion

This study suggests that symptoms of chronic neck pain and various eye symptoms in a cohort of patients with no known initiating event potentially share a common etiology: ligamentous cervical instability and cervical dysstructure. Ligamentous cervical instability explains the chronic “muscular” neck tension and pain felt by patients, and the compression of the carotid sheath at the level of the atlas with the resultant narrowing of the IJVs and vagus nerve degeneration. As it relates to the development of eye symptoms, the former leads to an increase in CSF pressure around the optic nerve and the latter contributes to ocular dysautonomia. The net effects of these causes are elevated ONSD, mydriasis (pupil dilation), abnormal pupillary light reflexes, and increased IOP, the combination of which could be responsible for the various eye symptoms experienced by this patient population, including blurry vision, eye pain/pressure, eye tearing, light sensitivity, seeing flashes of light, and visual changes.

## Data Availability

The raw data supporting the conclusions of this article will be made available by the authors, without undue reservation.
